# Effects of Lanthanum Doping on the Microstructure and Electromagnetic Properties of X-Type Hexaferrite Ba_2_Co_2_Fe_28_O_46_ Prepared by High-Temperature Solid-State Reaction

**DOI:** 10.3390/ma19132703

**Published:** 2026-06-23

**Authors:** Ning Li, Ziyu Guo, Yupeng Zhang, Qin Li, Fuyuan Dong, Gangli Feng

**Affiliations:** 1 School of Materials Science and Engineering, North Minzu University, Yinchuan 750021, China; wglining@163.com (N.L.); g20257389@163.com (Z.G.); grokcopper2025@163.com (Y.Z.); 18602799769@163.com (Q.L.); fydong@alum.imr.ac.cn (F.D.); 2 National and Local Joint Engineering Research Center of Advanced Carbon-Based Ceramics Preparation Technology, Yinchuan 750021, China

**Keywords:** X-type hexagonal ferrite, lanthanum-doping, high-temperature solid-state method, electromagnetic properties, microwave absorption

## Abstract

With the advancement of electronics and communication technologies, there is growing interest in high-performance microwave-absorbing materials. The material composition and structural design are critical factors influencing the electromagnetic wave (EMW) absorption capabilities. X-type barium ferrite (Ba_2_Co_2_Fe_28_O_46_) exhibits advantages in enhancing high-frequency magnetic loss and interface polarization through its unique hexagonal crystal structure and morphological design, while also optimizing impedance matching to a certain extent. However, the effective absorption bandwidth (EAB) of single-phase barium ferrite is often restricted. Therefore, doping with other elements is necessary to broaden the EAB. In this study, La^3+^-substituted X-type hexagonal ferrites Ba_2_Co_2_Fe_28−x_La_x_O_46_ (x = 0.00, 0.05, 0.10, 0.15, and 0.20) were successfully synthesized via a high-temperature solid-state reaction method, and the effects of different La^3+^ doping concentrations on the electromagnetic parameters and wave-absorbing performance of Ba_2_Co_2_Fe_28_O_46_ were investigated. After doping, the materials demonstrated excellent electromagnetic absorption performance: when x = 0.15, RL_min_ = −48.36 dB; when x = 0.10, EAB = 9.03 GHz (RL ≤ −5 dB).

## 1. Introduction

In recent years, with the rapid advancement of 5G technology, electronic communication devices incorporating RF chips have been widely adopted. However, while they are enjoyed for their convenience, the issue of electromagnetic radiation pollution cannot be overlooked [1]. In addition to affecting human health, the electromagnetic waves generated by electronic devices can easily cause electromagnetic interference with surrounding electronic equipment, leading to malfunctions or impaired signal transmission [2,3]. Consequently, the development of lightweight, broadband, and highly efficient microwave-absorbing materials has become a research focus. Among various candidates, hexagonal ferrites are regarded as highly promising absorbers owing to their natural uniaxial magnetocrystalline anisotropy, high chemical stability, and low-loss characteristics at high frequencies [4,5,6]. Specifically, X-type hexagonal ferrite (Ba_2_Me_2_Fe_28_O_46_), featuring a unique crystal structure formed by the alternate stacking of M-type and W-type blocks, offers a high cutoff frequency and moderate saturation magnetization. This endows it with good soft magnetic properties and impedance-matching potential in the GHz band [7,8,9].

However, preparing pure-phase X-type ferrite via traditional high-temperature solid-state methods is challenging, often resulting in impurities such as M-type and W-type phases that severely degrade intrinsic electromagnetic properties [10]. Ionic substitution is a core strategy for tuning the performance of hexagonal ferrites. Recently, rare-earth ion doping has become a research focus for optimizing the magnetoelectric properties of ferrites owing to its unique 4f electron configuration and large ionic radius [11,12,13]. Although reports exist on rare-earth (e.g., Ce, Nd, Y)-doped hexagonal ferrites [14,15,16], systematic studies on the impact of La^3+^ doping at Fe^3+^ sites in X-type ferrites remain insufficient. In particular, the structure–property relationships concerning microstrain and multi-scale electromagnetic responses in La^3+^-doped X-type ferrites prepared by high-temperature solid-state methods require further clarification.

Recent studies have indicated that rare-earth doping induces lattice distortion and modulates magnetocrystalline anisotropy by altering the Fe^3+^-O^2−^-Fe^3+^ superexchange pathways [17,18]. For instance, Dai, Y. et al. demonstrated that La-Co co-doping can simultaneously improve the permeability and dielectric loss tangent of M-type strontium hexaferrite, which is beneficial for enhancing microwave absorption. However, the article only discusses changes in permeability and dielectric loss, without addressing core application metrics such as microwave absorption performance, impedance matching, and the attenuation constant, thereby severing the intrinsic connection between electromagnetic parameters and wave-absorbing performance [19]. Kumar, P. et al. reported that La-Zn co-doping can effectively improve the dielectric, ferroelectric, magnetic and magnetoelectric coupling properties of M-type barium hexaferrite, which is conducive to the development of room-temperature multiferroic materials. However, this study only analyzes the structural, morphological, and basic electromagnetic parameters, without exploring key indicators such as the magnetoelectric coupling mechanism, spin structure evolution, and practical device performance, thus failing to clarify the essential relationship between doping modification and functional application potential [20]. Nevertheless, in X-type systems, the synergistic effects of lattice expansion, defect chemistry, and interface polarization induced by the large ionic radius of La^3+^ (1.06 Å), substituting for Fe^3+^ (0.645 Å), on the microwave absorption performance require deeper exploration. Furthermore, most existing studies concentrate on wet chemical methods such as sol–gel and citrate–nitrate combustion methods, while reports on the microstructure and electromagnetic property regulation mechanisms of products obtained via the industrially preferable high-temperature solid-state method are scarce [21,22].

To address these gaps, this study designed and prepared La^3+^-substituted Ba_2_Co_2_Fe_28−x_La_x_O_46_ series samples via a high-temperature solid-state reaction method. This substitution effectively introduces lattice distortion, leading to improved impedance matching and a broader absorption bandwidth.

## 2. Materials and Methods

Lanthanum oxide (La_2_O_3_, 99.99%) was purchased from Shanghai Titan Scientific Co., Ltd. (Shanghai, China). Barium carbonate (BaCO_3_, 99.0%) was obtained from Shanghai Guangnuo Chemical Technology Co., Ltd. (Shanghai, China). Ferric oxide (Fe_2_O_3_) was obtained from Shanghai Shanpu Chemical Co., Ltd. (Shanghai, China). Cobalt tetroxide (Co_3_O_4_, 99.9%) was obtained from Shanghai Macklin Biochemical Co., Ltd. (Shanghai, China). Anhydrous ethanol (C_2_H_6_O) was used as received.

The raw materials BaCO_3_, Co_3_O_4_, Fe_2_O_3_, and La_2_O_3_ were weighed according to the stoichiometric ratio, establishing five experimental groups: undoped and La^3+^ doping molar fractions of 0.05, 0.10, 0.15, and 0.20. The raw materials were placed in a ball-milling jar with anhydrous ethanol as the dispersing medium. Planetary ball milling was performed at 300 rpm (forward/reverse alternating) for 3 h. The slurry was then poured into glass vessels and left at room temperature to volatilize until completely dry.

**Figure 1:**
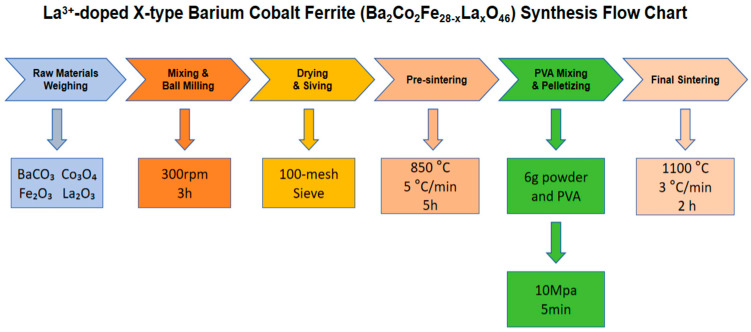
Ceramic sample processing workflow.

The dried powder was ground, loaded into alumina crucibles, and placed in a box-type resistance furnace for presintering. The furnace was calcined at 850 °C for 5 h with a heating ramp of 5 °C/min and then cooled naturally. The resulting powder was passed through a 100-mesh sieve to obtain presintered powder. Six grams of presintered powder was mixed with eight drops of Polyvinyl Alcohol (PVA; 5% wt). This mixture was uniformly pressed into disk-shaped specimens using a powder tablet press at 10 MPa for 5 min. Finally, Ba_2_Co_2_Fe_28−x_La_x_O_46_ (x = 0.00, 0.05, 0.10, 0.15, and 0.20) was sintered at 1100 °C for 2 h with a heating ramp of 3 °C/min, followed by cooling at 5 °C/min to 300 °C before furnace cooling to room temperature to yield the final La^3+^-doped X-type Ba_2_Co_2_Fe_28_O_46_ ferrite samples. The ceramic sample processing workflow is shown in Figure 1.

## 3. Results

### 3.1. Phase and Morphology Analysis

**Figure 2:**
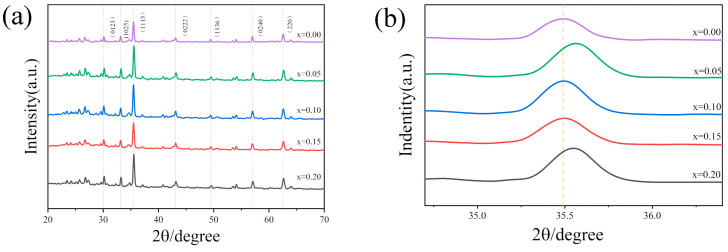
(**a**) XRD patterns of Ba_2_Co_2_Fe_28−x_La_x_O_46_ (x = 0.00, 0.05, 0.10, 0.15, 0.20); (**b**) magnified view of the characteristic (1115) diffraction peak. The data were processed and plotted using OriginPro 2026, which was downloaded from the official website of OriginLab (https://www.originlab.com, accessed on 10 May 2026).

Figure 2a shows the XRD patterns of samples with different La^3+^ doping levels. Characteristic diffraction peaks of the hexagonal ferrite phase appeared in all samples, exhibiting sharp peak shapes. The primary peaks located at 2θ = 30.1°, 32.16°, 35.49°, 43.1°, 44.34°, 56.94°, and 62.54° correspond to the (0123), (1025), (1115), (0222), (1136), (0240), and (220) crystal planes, respectively, accurately matching with the standard card (JCPDS No. 73-2034) [23]. Figure 2b clearly shows a shift in the main diffraction peak caused by different doping levels. At low doping (x = 0.05), a small amount of La^3+^ substituted for Fe^3+^, which instantly induced a small number of oxygen vacancies. The lattice contraction effect from these vacancies outweighs the lattice expansion caused by the La ions, resulting in a decrease in d-spacing and a slight rightward shift in the peak [24,25]. When x = 0.10 and 0.15, the lattice expansion caused by La^3+^ is counteracted by the lattice contraction from oxygen vacancies, effectively canceling each other out; thus, the interplanar spacing d remains essentially the same as that of the undoped sample, and the peak position remains constant [26]. At the doping level of x = 0.20, the doping exceeded the critical equilibrium threshold, and the lattice could no longer relieve the distortion through strain relaxation, leading to a reduction in the interplanar spacing d [24,25].

**Figure 3:**
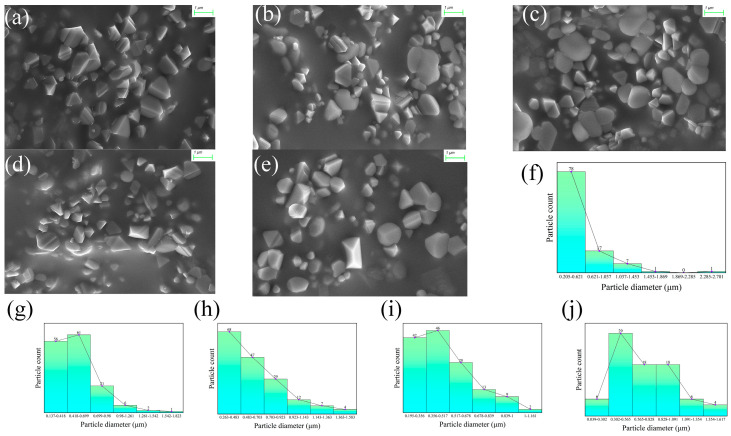
SEM images of (**a**–**e**) Ba_2_Co_2_Fe_28−x_La_x_O_46_ (x = 0.00, 0.05, 0.10, 0.15 and 0.20). Particle size statistics of (**f**–**j**) Ba_2_Co_2_Fe_28−x_La_x_O_46_ (x = 0.00, 0.05, 0.10, 0.15 and 0.20). The data were processed and plotted using OriginPro.

SEM images of the samples are shown in Figure 3. Figure 3a shows that the undoped sample (x = 0.00) exhibits irregular flake and block morphologies with a wide grain size distribution and significant agglomeration, typical characteristics of incomplete solid-state reactions and abnormal grain growth in high-temperature solid-state methods [27]. Figure 3c,d reveal that with the introduction of La^3+^ (x = 0.10 to 0.15), grain size gradually decreases, morphologies tend toward regular hexagonal flakes, and distribution becomes more uniform. This is attributed to the enrichment of lanthanide ions at the grain boundaries, which inhibits grain boundary migration, thereby refining the grains [27,28]. However, when the doping level increased to x = 0.20 (Figure 3e), signs of abnormal grain growth and slight melting appeared in some regions. This is caused by excess La^3+^ enrichment at the grain boundaries to form low-melting-point liquid phases, promoting mass transport [29]. These microstructural changes directly affect densification behavior and electromagnetic response.

Based on the SEM particle size histograms (Figure 3f–j) and Table 1 data, La doping significantly regulates the grain size and dimensional uniformity of Ba_2_Co_2_Fe_28−x_La_x_O_46_. The undoped sample (x = 0.00) displayed an average grain size of 0.540 μm and a standard deviation of 0.362 μm, featuring a broad particle size distribution and poor grain uniformity. At a low doping of x = 0.05, the average grain size remained nearly constant, while the standard deviation decreased to 0.263 μm, accompanied by a narrowed particle size distribution. When the doping level increases to x = 0.10, accumulated lattice distortion accelerates Ostwald ripening [30] and leads to grain coarsening up to 0.630 μm. When the doping content is x = 0.15, the segregation of La^3+^ at grain boundaries induces grain boundary pinning, refining the grains to the minimum average size of 0.491 μm among all specimens. Meanwhile, the standard deviation decreases to 0.201 μm, showing optimal particle size uniformity. At x = 0.20, the distribution evolves into a typical bimodal pattern with both small and large particle peaks coexisting, revealing severe agglomeration and abnormal growth under high-concentration-doped samples [31]. In summary, the particle size statistics align well with the SEM observations, confirming that appropriate La^3+^ doping effectively refines the grains, while excessive doping exceeds the solid solubility limit, triggering particle coarsening and bimodal distribution.

### 3.2. Electromagnetic Parameter Measurement

**Figure 4:**
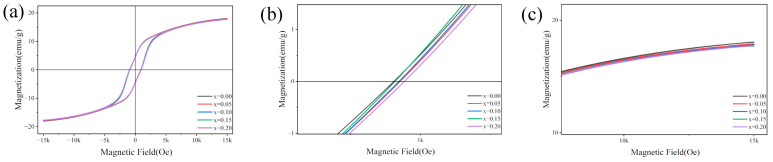
(**a**) Magnetic hysteresis loops of Ba_2_Co_2_Fe_28−x_La_x_O_46_ (x = 0.00, 0.05, 0.10, 0.15, and 0.20); (**b**) coercivity H_c_; (**c**) saturation magnetization M_s_. The data were processed and plotted using OriginPro.

The magnetic hysteresis loops were measured using a vibrating sample magnetometer (VSM), which showed that all samples exhibited typical ferromagnetic characteristics. In Figure 4a,b, the saturation magnetization M_s_ and remanent magnetization M_r_ generally exhibit a declining trend. This phenomenon is attributed to the substitution of magnetic Fe^3+^ by nonmagnetic La^3+^, which directly reduces the concentration of magnetic ions [32]. However, La^3+^-induced lattice distortion alters the Fe^3+^-O^2−^-Fe^3+^ bond angle, enhancing superexchange interactions between A–B sublattices, thereby increasing the net magnetic moment [33]. By observing changes in coercivity (H_c_), it is evident that La doping increases H_c_ from 905 Oe (x = 0.00) to 942 Oe (x = 0.20). This is attributed to the enhanced magnetocrystalline anisotropy induced by La^3+^, along with grain refinement and surface defects that increase domain wall pinning centers, thereby improving the material’s resistance to demagnetization [34]. The hysteresis loop parameters of the sample are shown in Table 2.

In this experiment, the coaxial air-line method was used to test the dielectric constant and permeability. The core principle involves fabricating the material into a toroidal shape embedded within a section of standard coaxial airline, using a Vector Network Analyzer (VNA) to measure electromagnetic parameters, from which the intrinsic electromagnetic parameters and microwave absorption performance are derived.

By testing the dielectric constant and permeability, the electromagnetic compatibility (EMA) performance of composites made of dielectric and magnetic materials can be characterized. According to electromagnetic field theory, the performance of microwave absorbers can be indirectly evaluated by studying the complex permittivity (ε_r_ = ε′ − jε″) and complex permeability (μ_r_ = μ′ − jμ″). The real parts (ε′ and μ′) reflect the storage capacity of the electromagnetic energy, whereas the imaginary parts (ε″ and μ″) are related to the dissipation of microwave energy [35].

**Figure 5:**
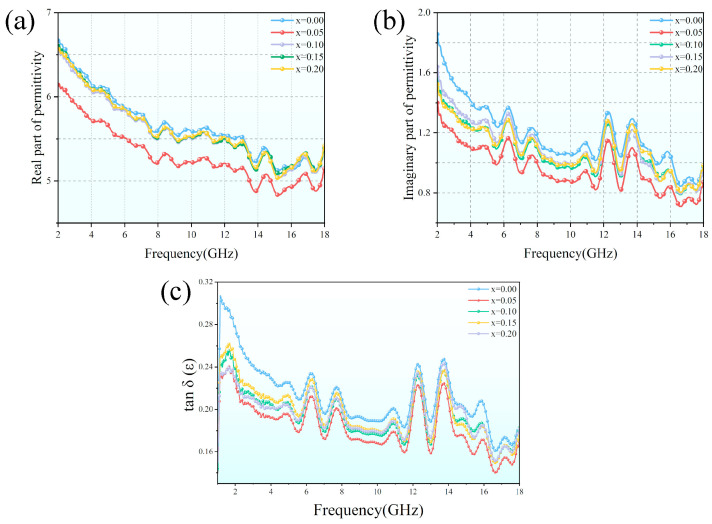
Real part (**a**) and imaginary part (**b**) of complex permittivity; (**c**) tan δ(ε). The data were processed and plotted using OriginPro.

Figure 5a clearly shows that ε′ for all samples decreases monotonically with increasing frequency, with the undoped sample exhibiting the highest ε′ across the entire band. As the La^3+^ content increases, ε′ decreases systematically because the ionic radius of La^3+^ (~1.03 Å) is much larger than that of Fe^3+^ (~0.645 Å). Substituting Fe^3+^ with La^3+^ causes lattice distortion, weakening the efficiency of ion displacement polarization. Simultaneously, the fully filled 4f electron shell of La^3+^ (no unpaired electrons) contributes far less to electronic polarization than Fe^3+^, leading to reduced overall polarization capability and thus lower ε′ [36,37].

As shown in Figure 5b, the ε″ curves of all samples exhibit distinct relaxation peaks within 2–18 GHz. These relaxation peaks are typical manifestations of electric dipoles, formed by lattice defects, dopant ions, or charge inhomogeneity at grain boundaries, undergoing relaxation in an alternating external electric field. The position and intensity of the relaxation peaks are related to the type, concentration, and relaxation time distribution of the dipoles [38,39]. La^3+^ doping can reduce lattice defects. In the undoped state, Fe^3+^ is easily reduced to Fe^2+^, forming defect dipoles that cause large fluctuations in ε″. After doping, the stabilizing effect of La^3+^ inhibits Fe valence fluctuations, reducing defect dipoles and lowering relaxation peak intensity. With increasing La^3+^ doping, the overall level of ε″ decreases, and the relaxation peaks become smoother and shift slightly toward higher frequencies, clearly indicating that La^3+^ doping effectively suppresses electron hopping and charge accumulation, reducing detrimental free charge movement and thus lowering dielectric loss. Based on Debye theory, the polarization relaxation process was further analyzed using the following equations [40]:

(1) $$\varepsilon^{\prime }={\;\varepsilon }_{\infty }+\frac{\varepsilon_{s}\;-\;\varepsilon_{\infty }}{1\;+\;\omega^{2}\tau^{2}}$$

(2) $$\varepsilon^{\prime \prime }={\varepsilon^{\prime \prime }}_{p}+{\varepsilon^{\prime \prime }}_{c}=\frac{\omega \tau (\varepsilon_{s}-\varepsilon_{\infty })}{1+{\;\omega }^{2}\tau^{2}}+\frac{\sigma }{\omega \varepsilon_{0}}$$

(3) $${\left(\varepsilon^{\prime }-\frac{\varepsilon_{s}+{\;\varepsilon }_{\infty }}{2}\right)}^{2}+{\left(\varepsilon^{\prime \prime }\right)}^{2}={\;(\frac{\varepsilon_{s}-\varepsilon_{\infty }}{2})}^{2}$$

where $\varepsilon_{s}$ and $\varepsilon_{\infty }$ are the static and infinite-frequency dielectric constants, respectively; ε_0_ represents the vacuum permittivity; τ denotes the polarization relaxation time; ω is the angular frequency; and σ signifies conductivity. Figure 5c shows that the dielectric loss tangent (tan δ) generally decreases as the x increases from 0.00 to 0.20, particularly in the low-frequency range. This demonstrates that doping can effectively reduce the dielectric loss of a material.

**Figure 6:**
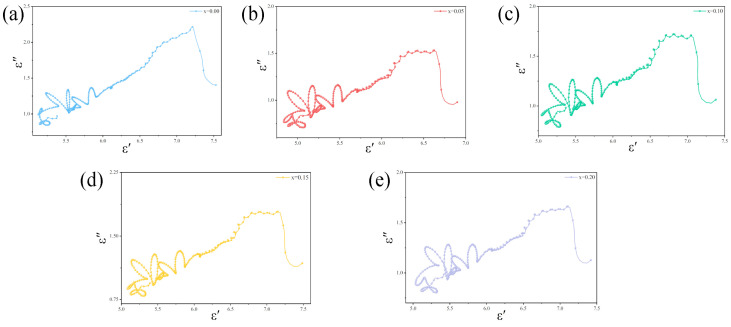
Cole–Cole diagram of Ba_2_Co_2_Fe_28−x_La_x_O_46_. (**a**) x = 0.00; (**b**) x = 0.05; (**c**) x = 0.10; (**d**) x = 0.15; (**e**) x = 0.20. The data were processed and plotted using OriginPro.

Figure 6 further illustrates the influence of the doping content on the conduction and polarization losses of the material. When the curve between ε′ and ε″ is a semicircle, each semicircle corresponds to a Debye relaxation process [41]. The undoped sample exhibited multiple spiral-shaped semicircular arcs, reflecting the coexistence of multiple polarization-relaxation processes. As the doping content increased, the number, amplitude, and rising slope of the tail of the semicircular arcs gradually decreased, indicating that doping simultaneously suppressed both polarization relaxation and conduction loss.

**Figure 7:**
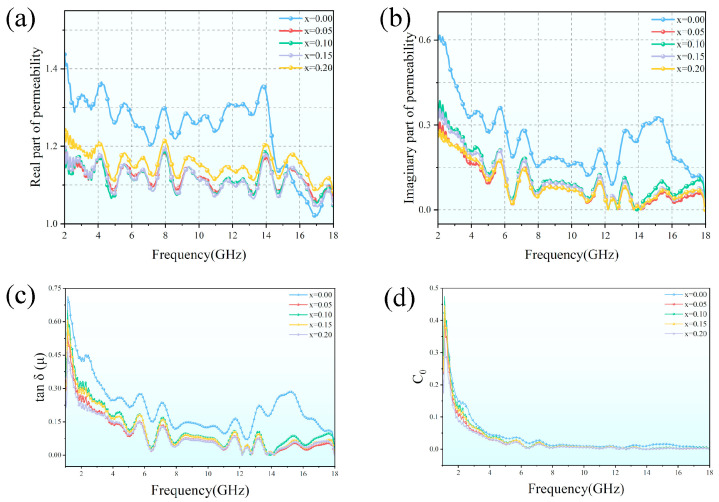
Real part (**a**) and imaginary part (**b**) of complex permeability; (**c**) magnetic loss tangent (tanδμ); (**d**) C_0_. The data were processed and plotted using OriginPro.

In Figure 7a, the μ′ of the undoped sample exhibits intense multifrequency oscillations, indicating complex magnetization processes and high-intensity resonance modes. Upon introducing La^3+^ (from x = 0.05 to 0.20), the oscillation amplitude weakened noticeably, and the overall trend became smoother. This is because La^3+^ doping significantly alters the magnetocrystalline anisotropy field. As a nonmagnetic ion, La^3+^ entering the lattice dilutes the superexchange interactions between magnetic ions (Fe^3+^, Co^2+^) and adjusts the magnetocrystalline anisotropy constant by changing the crystal field environment [42].

Figure 7b shows the μ″ curves, which visually display the position and intensity of magnetic loss peaks. μ″ reflects magnetic loss (e.g., eddy current loss, hysteresis loss, natural resonance loss). In the undoped state, lattice defects (oxygen vacancies, Fe^2+^) lead to large eddy current and hysteresis losses, causing drastic μ″ fluctuations; after La^3+^ doping, defects decrease, eddy current loss is reduced, and the magnetocrystalline anisotropy field weakens, causing drastic μ″ fluctuations [43,44]. All samples exhibited a main loss peak at 4–6 GHz and a secondary peak near 12–16 GHz. This confirms the existence of a multiresonance mechanism, where the low-frequency main peak is typically attributed to domain wall resonance, and the high-frequency secondary peak likely corresponds to natural ferromagnetic resonance or exchange resonance. At x = 0.00, the main loss peak is located near 5 GHz, corresponding to the strongest capability of magnetic moments to convert electromagnetic energy into heat at the resonance frequency. With La^3+^ doping, the main loss peak shifted toward higher frequencies. This phenomenon can be further explained by the following formulas [39]:

(4) $$\mu^{\prime }\;=\;1\;+\;(M/H)\cos\sigma$$

(5) $$\mu^{\prime \prime }=1+(M/H)\sin\sigma$$

where M represents the magnetization (vector sum of the magnetic dipole moments per unit volume), H denotes the applied magnetic field strength, and σ is the angle between the magnetization vector M and applied field H.

Figure 7c clearly illustrates the inhibitory effect of frequency on the magnetic loss tangent: the loss decreases with an increase in frequency. The undoped material exhibited the highest loss. As the doping content increased, the value of tanδ decreased and tended to be stable, reaching its minimum at x = 0.20. This indicates that doping significantly weakens hysteresis and eddy current losses.

Typically, the magnetic loss mechanism of a material originates from eddy current loss, exchange resonances and resonance loss [44]. C_0_ is used to characterize the magnetic loss mechanism and calculated via the following formula [45]:

(6) $$C_{0}\;=\;\mu^{\prime \prime }{\left(\mu^{\prime }\right)}^{-2}f^{\;-1}$$

When the C_0_ value does not change with frequency, it indicates that the magnetic loss is primarily due to eddy current loss. In contrast, when C_0_ changes, it indicates that the eddy current loss is very weak [46]. Figure 7d shows the frequency-dependent C_0_ curves of the samples with different doping contents. The C_0_ values of all samples decreased significantly within the range of 2–6 GHz and then tended to be stable in the 6–18 GHz band, indicating that magnetic loss was mainly dominated by hysteresis and resonance relaxation processes at low frequencies, whereas eddy current loss became the main contributor at high frequencies. With an increase in the doping content, the overall level of C_0_ decreased and stabilized earlier, demonstrating that doping effectively weakened the eddy current loss of the material, which is consistent with the variation in the magnetic loss tangent.

α is a characteristic parameter that describes how rapidly the amplitude of electromagnetic waves decays with propagation distance inside a medium. Determined jointly by the complex permittivity and complex permeability of materials, it serves as a core physical quantity for evaluating the electromagnetic wave loss capability [47].

(7) $$\alpha =\frac{\sqrt{2}\pi f}{c}\sqrt{(\mu^{\prime \prime }\varepsilon^{\prime \prime }\;-\;\mu^{\prime }\varepsilon^{\prime })+\sqrt{{\left(\mu^{\prime }\varepsilon^{\prime \prime }+\mu^{\prime }\varepsilon^{\prime }\right)}^{2}+{\left(\mu^{\prime \prime }\varepsilon^{\prime \prime }\;-\;\mu^{\prime }\varepsilon^{\prime }\right)}^{2}}}$$

**Figure 8:**
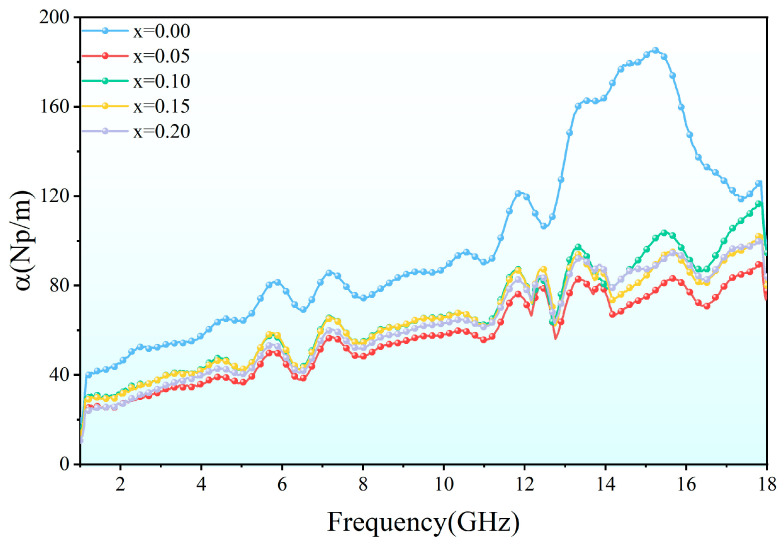
Attenuation factor (α). The data were processed and plotted using OriginPro.

Figure 8 shows the frequency dependence of the attenuation constant (α) for samples with different doping contents. The α values of all the samples increased with frequency, indicating stronger electromagnetic wave attenuation at higher frequencies. The undoped sample (x = 0.00) exhibited the highest α across the entire frequency range, reaching a peak of ~185 Np/m at 15–16 GHz, demonstrating the strongest electromagnetic wave attenuation capability. With the introduction of dopants, the α-values generally decreased. The sample with x = 0.05 shows the lowest attenuation constant, while those with x = 0.10, 0.15 and 0.20 show a slight recovery in α, but it still remains lower than that of the undoped sample. These results reveal that doping weakens the overall attenuation ability of the material.

### 3.3. Microwave Absorption Performance Testing

Reflection Loss (RL) is one of the key parameters determining electromagnetic wave (EMW) absorption performance. An RL exceeding −5 dB indicates that approximately 68% of incident waves can be effectively absorbed. According to classical transmission line theory, the RL value can be expressed by the following formulas [44,48]:

(8) $$\mathrm{RL}\;\left(\mathrm{dB}\right)\;=\;20\log_{10}\frac{|Z_{\mathrm{in}}\;-\;1|}{|Z_{\mathrm{in}}\;+\;1|}$$

(9) $$Z_{\mathrm{in}}={\;Z}_{0}\sqrt{\frac{\mu_{r}}{\varepsilon_{r}}}\tan h[j\frac{\omega d}{c}\sqrt{\mu_{r}\varepsilon_{r}}]$$

where ε_r_ is the complex permittivity; µ_r_ is the complex permeability; Z_in_ is the input impedance; and d and c are the thickness and speed of light, respectively.

**Figure 9:**
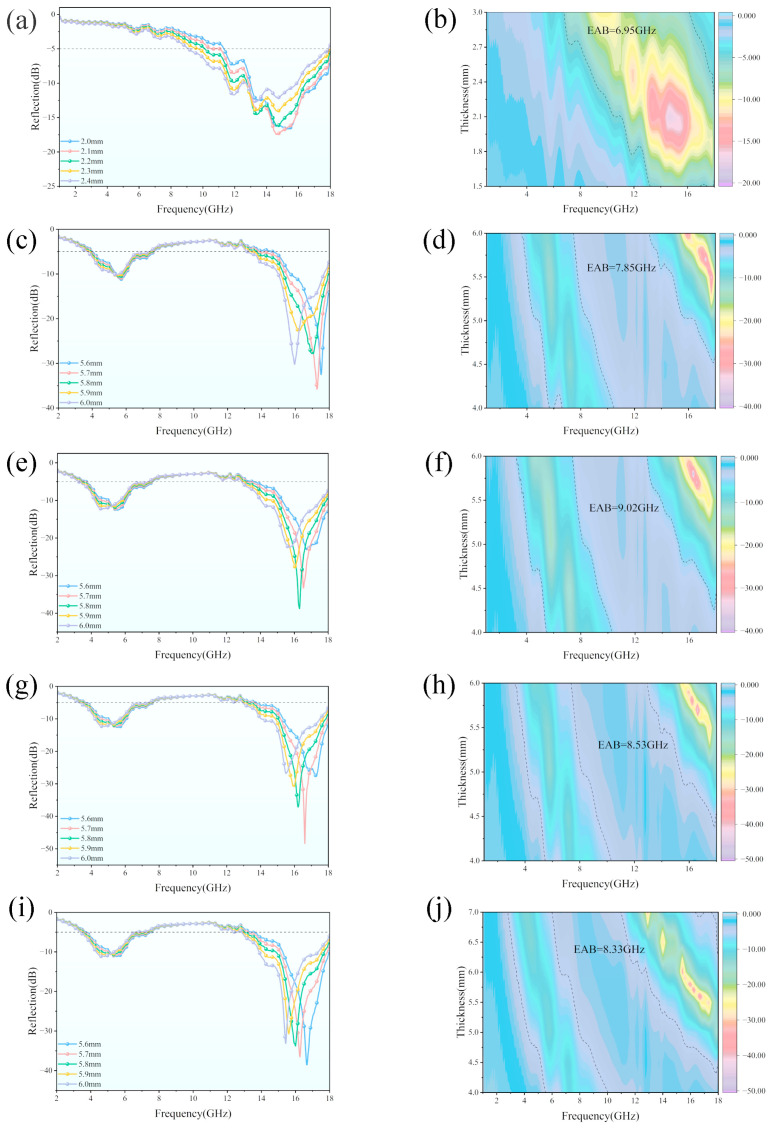
Reflection loss curves of samples with doping amount: (**a**,**b**) x = 0.00; (**c**,**d**) x = 0.05; (**e**,**f**) x = 0.10; (**g**,**h**) x = 0.15; (**i**,**j**) x = 0.20. The data were processed and plotted using OriginPro.

According to the quarter-wavelength matching model shown in Figure 9, impedance matching of electromagnetic waves inside the absorber follows the equation [49]:

(10) $$t_{m}\;=\;\frac{n\lambda }{4}\;=\;\frac{nc}{4f_{m}\sqrt{\left|\mu_{r}\varepsilon_{r}\right|}}\;(n\;=\;1,\;3,\;5\ldots )$$

where t_m_ represents the thickness of the absorbers, λ denotes the wavelength, c is considered the speed of light in a vacuum, f_m_ signifies the frequency at the peak, |µ_r_| and |ε_r_| stand for the moduli of the ε_r_ and µ_r_ at f_m_, respectively, and n is an odd number (1, 3, 5…).

**Figure 10:**
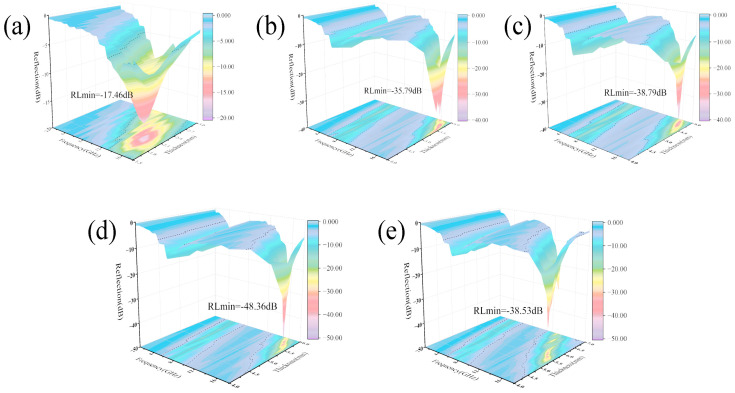
3D reflection loss diagram (**a**) x = 0.00; (**b**) x = 0.05; (**c**) x = 0.10; (**d**) x = 0.15; (**e**) x = 0.20. The data were processed and plotted using OriginPro.

To verify the microwave absorption performance of Ba_2_Co_2_Fe_28−x_La_x_O_46_ (x = 0.00, 0.05, 0.10, 0.15, and 0.20), RL curves were plotted based on the measured dielectric constants and permeabilities. Figure 9a–j illustrate the absorption performance from x = 0.00 to 0.20. Figure 9a shows that for the undoped sample, RL_min_ = −17.46 dB and EAB_max_ = 6.95 GHz (RL ≤ −5dB). Figure 9h–j (x = 0.15) demonstrate the excellent absorption intensity at a thickness of 5.7 mm, with RL_min_ = −48.36 dB. Figure 9e,f (x = 0.10) exhibit the best absorption bandwidth, EAB = 9.03 GHz, at a thickness of 5.8 mm. Figure 10 shows the 3D reflection loss diagram.A comparison between this work and other relevant literatures is presented in Table 3.

## 4. Conclusions

This study successfully fabricated X-type hexagonal ferrite Ba_2_Co_2_Fe_28−x_La_x_O_46_ using a high-temperature solid-phase reaction method, where La^3+^ replaced Fe^3+^ sites. Structural characterization and electromagnetic performance tests demonstrated that the introduction of La^3+^ induces significant lattice distortion and optimizes the material’s intrinsic electromagnetic properties. This microstructural evolution effectively enhanced the system’s impedance-matching performance and substantially broadened the effective absorption bandwidth. Experimental results reveal outstanding microwave absorption performance: at x = 0.15 and a matching thickness of 5.7 mm, the minimum reflection loss (RL_min_) reaches −48.36 dB, while at x = 0.10 and a thickness of 5.8 mm, the effective absorption bandwidth (EAB, RL ≤ −5 dB) extends up to 9.03 GHz. This study confirms that La^3+^ doping is an effective strategy to overcome the absorption bandwidth limitations of X-type ferrites, providing crucial experimental evidence for developing lightweight, wide-band microwave absorption materials for high-frequency applications.

## Figures and Tables

**Table 1:** Particle size analysis results for each sample, including total particle count, average particle size, and particle size standard deviation.

Sample	Total Particles	Average Particle Size (μm)	Particle Size Standard Deviation (μm)
x = 0.00	104	0.540	0.362
x = 0.05	147	0.540	0.263
x = 0.10	167	0.630	0.279
x = 0.15	140	0.491	0.201
x = 0.20	81	0.690	0.328

**Table 2:** Magnetic properties of the synthesized samples, including saturation magnetization (M_s_), remanent magnetization (M_r_), and coercivity (H_c_).

Sample	M_s_ (emu/g)	M_r_ (emu/g)	H_c_ (Oe)
x = 0.00	18.05	4.55	905
x = 0.05	17.89	4.50	927
x = 0.10	17.79	4.50	923
x = 0.15	17.72	4.54	908
x = 0.20	17.74	4.51	942

**Table 3:** Comparison of absorption performance between the results of different studies and the present work.

Sample	RL_min_ (dB)	Bandwidth (GHz)	Thickness (mm)	Reference
Ba_2.7_La_0.3_Co_2_Fe_24_O_41_	−47.3	4.32	3.5	[50]
Ba_2_Co_2_Ce_0.1_Fe_27.9_O_46_	−51.2	5.8	2.9	[51]
Ba_0.95_La_0.05_Co_2_Fe_15.5_Al_0.5_O_27_	−50.13	1.7	7.0	[52]
Ba_2_Co_2_Fe_27.9_La_0.1_O_46_	−38.79	9.03	5.8	This work
Ba_2_Co_2_Fe_27.85_La_0.15_O_46_	−48.36	8.53	5.7	This work

## Data Availability

The original contributions presented in this study are included in the article. Further inquiries can be directed to the corresponding author.
